# Tris(ethyl­enediamine)­cobalt(III) diformatodioxalatoindate(III) dihydrate

**DOI:** 10.1107/S1600536811013109

**Published:** 2011-04-13

**Authors:** Juying Tong, Qinhe Pan

**Affiliations:** a School of Materials Science and Engineering, Shanghai University, Shanghai 201800, People’s Republic of China; b Department of Materials and Chemical Engineering, Ministry of Education Key Laboratory of Application Technology of Hainan, Superior Resources Chemical Materials, Hainan University, Haikou 570228, Hainan Province, People’s Republic of China

## Abstract

In the cation of the title compound, [Co(C_2_H_8_N_2_)_3_][In(C_2_O_4_)_2_(CHO_2_)_2_]·2H_2_O, the Co—N bond lengths lie in the range 1.960 (5)–1.997 (5) Å. In the anion, the In^III^ atom is coordin­ated by four O atoms from two oxalato ligands and two O atoms from two formato ligands in a distorted octa­hedral geometry. Inter­molecular O—H⋯O and N—H⋯O hydrogen bonds form an extensive hydrogen-bonding network, which link the cations, anions and water mol­ecules into three-dimensional structure.

## Related literature

For related structures, see: Chen *et al.* (2005 ▶); Du *et al.* (2004 ▶); Pan *et al.* (2005 ▶, 2008 ▶, 2010*a* ▶,*b* ▶, 2011 ▶); Stalder & Wilkinson (1997 ▶); Wang *et al.* (2003*a* ▶,*b* ▶,*c* ▶, 2004 ▶); Yu *et al.* (2001 ▶); Zhang *et al.* (2003*a* ▶,*b* ▶). 
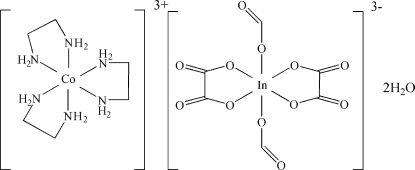


## Experimental

### 

#### Crystal data

[Co(C_2_H_8_N_2_)_3_][In(C_2_O_4_)_2_(CHO_2_)_2_]·2H_2_O
*M* *_r_* = 656.17
Triclinic, 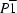

*a* = 8.2048 (16) Å
*b* = 12.016 (2) Å
*c* = 12.052 (2) Å
α = 79.09 (3)°
β = 81.45 (3)°
γ = 88.43 (3)°
*V* = 1153.7 (4) Å^3^
*Z* = 2
Mo *K*α radiation
μ = 1.80 mm^−1^
*T* = 293 K
0.2 × 0.18 × 0.15 mm

#### Data collection

Rigaku R-AXIS RAPID-S diffractometer
Absorption correction: multi-scan (*CrystalClear*; Rigaku/MSC, 2002 ▶) *T*_min_ = 0.686, *T*_max_ = 1
12132 measured reflections
5263 independent reflections
3963 reflections with *I* > 2σ(*I*)
*R*_int_ = 0.084

#### Refinement

*R*[*F* ^2^ > 2σ(*F* ^2^)] = 0.060
*wR*(*F* ^2^) = 0.168
*S* = 1.05
5263 reflections
311 parameters
H-atom parameters constrained
Δρ_max_ = 1.01 e Å^−3^
Δρ_min_ = −1.13 e Å^−3^

### 

Data collection: *RAPID-AUTO* (Rigaku, 1998 ▶); cell refinement: *RAPID-AUTO*; data reduction: *CrystalClear* (Rigaku/MSC, 2002 ▶); program(s) used to solve structure: *SHELXS97* (Sheldrick, 2008 ▶); program(s) used to refine structure: *SHELXL97* (Sheldrick, 2008 ▶); molecular graphics: *SHELXTL* (Sheldrick, 2008 ▶); software used to prepare material for publication: *SHELXL97*.

## Supplementary Material

Crystal structure: contains datablocks I, global. DOI: 10.1107/S1600536811013109/cv5068sup1.cif

Structure factors: contains datablocks I. DOI: 10.1107/S1600536811013109/cv5068Isup2.hkl

Additional supplementary materials:  crystallographic information; 3D view; checkCIF report

## Figures and Tables

**Table 1:** Hydrogen-bond geometry (Å, °)

*D*—H⋯*A*	*D*—H	H⋯*A*	*D*⋯*A*	*D*—H⋯*A*
N1—H1*A*⋯O7^i^	0.90	2.22	3.019 (7)	148
N1—H1*B*⋯O6^ii^	0.90	2.25	2.981 (7)	139
N2—H2*A*⋯O1*W*^iii^	0.90	2.02	2.857 (7)	155
N2—H2*B*⋯O4^iv^	0.90	2.14	3.017 (6)	166
N3—H3*A*⋯O2*W*^v^	0.90	2.23	3.017 (7)	146
N3—H3*B*⋯O6^ii^	0.90	2.17	3.002 (7)	154
N3—H3*B*⋯O8^ii^	0.90	2.50	3.153 (7)	130
N4—H4*A*⋯O2	0.90	2.11	2.915 (6)	148
N4—H4*B*⋯O10^iv^	0.90	2.33	3.102 (7)	144
N4—H4*B*⋯O2^iv^	0.90	2.53	3.166 (7)	128
N5—H5*A*⋯O8^i^	0.90	2.16	2.986 (6)	153
N5—H5*B*⋯O4^iv^	0.90	2.27	3.022 (6)	141
N5—H5*B*⋯O2^iv^	0.90	2.28	3.060 (6)	145
N6—H6*A*⋯O1	0.90	2.05	2.917 (7)	161
N6—H6*B*⋯O6^ii^	0.90	2.19	3.024 (7)	154
O2*W*—H2*WA*⋯O3^vi^	0.55	2.36	2.889 (6)	163
O2*W*—H2*WB*⋯O12^vii^	0.55	2.22	2.751 (7)	166
O1*W*—H1*WA*⋯O2*W*	0.80	2.03	2.821 (7)	169
O1*W*—H1*WB*⋯O4	0.80	2.09	2.836 (6)	156

Symmetry codes: (i) 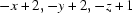; (ii) 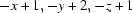; (iii) 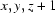; (iv) 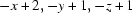; (v) 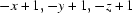; (vi) 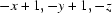; (vii) 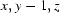.

**Fig. 1.**
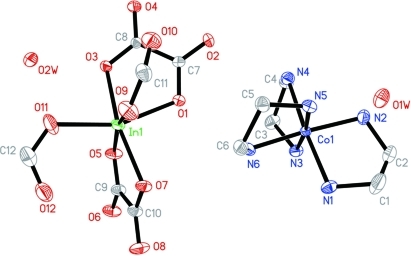
The content of asymmetric part of (I). Displacement ellipsoids are drawn at the 30% probability level. H atoms omitted for clarity.

## supplementary crystallographic information

### Comment

Template synthesis is an important method to get new materials. One of the interesting strategies is employing chiral metal complexes as a template, because they are versatile and can be made with a wide of shapes, charges and particularly chirality. Up to now, aluminophosphates such as [d-Co(en)_3_]Al_3_P_4_O_16_.2H_2_O (Stalder *et al.*, 1997) and [d-Co(en)_3_]AlP_2_O_8_.6.5H_2_O (Chen *et al.*, 2005), gallium phosphates such as [d-Co(en)_3_][H_3_Ga_2_P_4_O_16_] (Stalder *et al.*, 1997) and [Co(en)_3_][Ga_3_(H_2_PO_4_)_6_(HPO_4_)_3_] (Wang *et al.*, 2003*a*), zinc phosphates such as [Co(en)_3_][Zn_8_P_6_O_24_Cl].2H_2_O (Yu *et al.*, 2001) and [Co(dien)_2_.H_3_O][Zn_2_(HPO_4_)_4_] (Wang *et al.*, 2003*b*), indium phosphate [Co(en)_3_][In_3_(H_2_PO_4_)_6_(HPO_4_)_3_].H_2_O (Du *et al.*, 2004), germanates such as [Ni(1,2-PDA)_3_]_2_(HOCH_2_CH_2_CH_2_NH_3_)_3_(H_3_O)_2_[Ge_7_O_14_*X*_3_]_3_ (*X* = F, OH) (Pan *et al.*, 2008) and [Ni(dien)_2_]_2_[GeO_7_O_13_(OH)_2_F_3_]Cl (Zhang *et al.*, 2003*a*), and fluorogermanates such as [Ni(dien)_2_][GeF_6_] (Zhang *et al.*, 2003*b*), [Ni(en)(TETA)][GeF_6_] (Wang *et al.*, 2004), and [Ni(en)_3_][GeF_6_] (Pan *et al.*, 2005), have been reported. Also a new concept of chirality transfer of the metal complex into the inorganic host framework has been demonstrated (Wang *et al.*, 2003*c*). Recently, we reported some metal oxalate using metal complex cations as template (Pan *et al.*, 2010*a*,*b*, 2011). When formate anions were introduced to the system, the title compound (I) - a formate oxalate mixed coordinated complex - was obtained.

The crystal structure of (I) consists of a discrete [In(C_2_O_4_)_2_(HCO_2_)_2_]^3-^ anions and [Co(en)_3_]^3+^ cations (Fig. 1). The In(iii) ion is coordinated by two formate anions in a monodentate mode and two oxalate anions. The asymmetric part contains two crystalline water molecules. Intermolecular O—H···O and N—H···O hydrogen bonds (Table 1) form an extensive hydrogen-bonding network, which link cations, anions and crystalline water molecules into three-dimensional crystal structure.

### Experimental

In a typical synthesis, a mixture of In(NO_3_)_3_.5H_2_O (1 mmol), Co(en)_3_Cl_3_ (0.43 mmol), K_2_C_2_O_4_.H_2_O (2 mmol) and DMF (10 ml), was added to a 20 ml Teflon-lined reactor under autogenous pressure at 120 °C for 3 days.

### Refinement

C- and N-bound H atoms were positioned geometrically (C—H = 0.97Å;  N—H = 0.90 Å), and refined as riding, with *U*_iso_(H) = 1.2*U*_eq_(parent atom).. The water' H atoms were located in a difference map, and refined as riding with as found O—H bond lengths, and with *U*_iso_(H) fixed to 0.08 Å^-2^.

### Crystal data

**Table:**

[Co(C_2_H_8_N_2_)_3_][In(CHO_2_)_2_(C_2_O_4_)_2_]·2H_2_O	*Z* = 2
*M**_r_* = 656.17	*F*(000) = 664
Triclinic, *P*1	*D*_x_ = 1.889 Mg m^−^^3^
*a* = 8.2048 (16) Å	Mo *K*α radiation, λ = 0.71073 Å
*b* = 12.016 (2) Å	Cell parameters from 11095 reflections
*c* = 12.052 (2) Å	θ = 3.1–27.5°
α = 79.09 (3)°	µ = 1.80 mm^−^^1^
β = 81.45 (3)°	*T* = 293 K
γ = 88.43 (3)°	Block, yellow
*V* = 1153.7 (4) Å^3^	0.2 × 0.18 × 0.15 mm

### Data collection

**Table:**

Rigaku R-AXIS RAPID-S diffractometer	5263 independent reflections
Radiation source: fine-focus sealed tube	3963 reflections with *I* > 2σ(*I*)
graphite	*R*_int_ = 0.084
ω scans	θ_max_ = 27.5°, θ_min_ = 3.1°
Absorption correction: multi-scan (*CrystalClear*; Rigaku/MSC, 2002)	*h* = −10→10
*T*_min_ = 0.686, *T*_max_ = 1	*k* = −15→15
12132 measured reflections	*l* = −15→15

### Refinement

**Table:**

Refinement on *F*^2^	Primary atom site location: structure-invariant direct methods
Least-squares matrix: full	Secondary atom site location: difference Fourier map
*R*[*F*^2^ > 2σ(*F*^2^)] = 0.060	Hydrogen site location: inferred from neighbouring sites
*wR*(*F*^2^) = 0.168	H-atom parameters constrained
*S* = 1.05	*w* = 1/[σ^2^(*F*_o_^2^) + (0.0731*P*)^2^ + 0.3428*P*] where *P* = (*F*_o_^2^ + 2*F*_c_^2^)/3
5263 reflections	(Δ/σ)_max_ = 0.002
311 parameters	Δρ_max_ = 1.01 e Å^−^^3^
0 restraints	Δρ_min_ = −1.13 e Å^−^^3^

### Special details

**Table:**

Geometry. All e.s.d.'s (except the e.s.d. in the dihedral angle between two l.s. planes) are estimated using the full covariance matrix. The cell e.s.d.'s are taken into account individually in the estimation of e.s.d.'s in distances, angles and torsion angles; correlations between e.s.d.'s in cell parameters are only used when they are defined by crystal symmetry. An approximate (isotropic) treatment of cell e.s.d.'s is used for estimating e.s.d.'s involving l.s. planes.
Refinement. Refinement of *F*^2^ against ALL reflections. The weighted *R*-factor *wR* and goodness of fit *S* are based on *F*^2^, conventional *R*-factors *R* are based on *F*, with *F* set to zero for negative *F*^2^. The threshold expression of *F*^2^ > σ(*F*^2^) is used only for calculating *R*-factors(gt) *etc*. and is not relevant to the choice of reflections for refinement. *R*-factors based on *F*^2^ are statistically about twice as large as those based on *F*, and *R*- factors based on ALL data will be even larger.

### Fractional atomic coordinates and isotropic or equivalent isotropic displacement parameters (Å^2^)

**Table:**

	*x*	*y*	*z*	*U*_iso_*/*U*_eq_	
In1	0.74015 (5)	0.84824 (3)	0.22384 (3)	0.03043 (16)	
Co1	0.91881 (8)	0.71299 (6)	0.70020 (6)	0.02243 (19)	
O1	0.7451 (5)	0.7212 (3)	0.3813 (3)	0.0322 (9)	
O2	0.7964 (6)	0.5351 (3)	0.4313 (3)	0.0427 (11)	
O3	0.6793 (5)	0.6899 (3)	0.1777 (3)	0.0311 (9)	
O4	0.7309 (5)	0.5043 (3)	0.2225 (3)	0.0332 (9)	
O5	0.4826 (5)	0.8852 (4)	0.2719 (4)	0.0393 (10)	
O6	0.3200 (5)	1.0247 (4)	0.3203 (4)	0.0456 (11)	
O7	0.7497 (5)	0.9860 (3)	0.3147 (4)	0.0343 (9)	
O8	0.5935 (5)	1.1319 (4)	0.3600 (4)	0.0507 (12)	
O9	1.0041 (5)	0.8544 (4)	0.1954 (4)	0.0500 (12)	
O10	1.0526 (6)	0.6669 (4)	0.2194 (4)	0.0539 (13)	
O11	0.7412 (7)	0.9122 (5)	0.0455 (4)	0.0629 (15)	
O12	0.6375 (8)	1.0792 (5)	0.0718 (5)	0.0687 (16)	
N1	0.9683 (6)	0.8481 (4)	0.7639 (4)	0.0351 (12)	
H1A	1.0430	0.8927	0.7141	0.080*	
H1B	0.8760	0.8887	0.7769	0.080*	
N2	0.9620 (6)	0.6240 (5)	0.8484 (4)	0.0360 (12)	
H2A	0.8827	0.5714	0.8749	0.080*	
H2B	1.0592	0.5880	0.8388	0.080*	
N3	0.6829 (6)	0.7226 (4)	0.7557 (4)	0.0343 (11)	
H3A	0.6647	0.7027	0.8324	0.080*	
H3B	0.6474	0.7941	0.7362	0.080*	
N4	0.8610 (6)	0.5716 (4)	0.6530 (4)	0.0336 (11)	
H4A	0.8704	0.5827	0.5764	0.080*	
H4B	0.9314	0.5162	0.6759	0.080*	
N5	1.1499 (6)	0.7020 (4)	0.6257 (4)	0.0307 (11)	
H5A	1.2164	0.7433	0.6552	0.080*	
H5B	1.1837	0.6294	0.6383	0.080*	
N6	0.8846 (6)	0.8113 (4)	0.5550 (4)	0.0342 (11)	
H6A	0.8290	0.7732	0.5151	0.080*	
H6B	0.8245	0.8722	0.5692	0.080*	
C1	1.0354 (12)	0.8056 (8)	0.8742 (6)	0.072 (3)	
H1C	1.1543	0.7986	0.8582	0.080*	
H1D	1.0109	0.8604	0.9241	0.080*	
C2	0.9658 (11)	0.6976 (7)	0.9314 (7)	0.067 (2)	
H2C	0.8548	0.7081	0.9688	0.080*	
H2D	1.0315	0.6629	0.9893	0.080*	
C3	0.5917 (7)	0.6445 (6)	0.7038 (6)	0.0427 (16)	
H3C	0.5784	0.6795	0.6262	0.080*	
H3D	0.4832	0.6283	0.7477	0.080*	
C4	0.6898 (7)	0.5366 (5)	0.7039 (5)	0.0371 (14)	
H4C	0.6872	0.4946	0.7813	0.080*	
H4D	0.6452	0.4892	0.6590	0.080*	
C5	1.1587 (7)	0.7451 (5)	0.5007 (5)	0.0359 (14)	
H5C	1.1213	0.6875	0.4637	0.080*	
H5D	1.2711	0.7656	0.4667	0.080*	
C6	1.0470 (8)	0.8487 (5)	0.4871 (5)	0.0369 (14)	
H6C	1.0917	0.9098	0.5157	0.080*	
H6D	1.0360	0.8749	0.4074	0.080*	
C7	0.7596 (7)	0.6186 (5)	0.3633 (5)	0.0296 (12)	
C8	0.7208 (6)	0.6011 (4)	0.2445 (4)	0.0235 (11)	
C9	0.4559 (7)	0.9783 (5)	0.3048 (5)	0.0351 (14)	
C10	0.6120 (7)	1.0395 (5)	0.3296 (5)	0.0351 (14)	
C11	1.0973 (8)	0.7662 (7)	0.2032 (6)	0.0447 (16)	
H11A	1.2100	0.7789	0.1957	0.080*	
C12	0.6927 (10)	1.0096 (7)	0.0090 (7)	0.057 (2)	
H12A	0.6974	1.0326	−0.0696	0.080*	
O2W	0.5103 (6)	0.2921 (4)	0.0086 (4)	0.0538 (15)	
H2WA	0.482	0.2861	−0.028	0.080*	
H2WB	0.531	0.251	0.030	0.080*	
O1W	0.7148 (6)	0.4807 (5)	−0.0054 (4)	0.0612 (16)	
H1WA	0.668	0.422	−0.0017	0.080*	
H1WB	0.6890	0.4882	0.060	0.080*	

### Atomic displacement parameters (Å^2^)

**Table:**

	*U*^11^	*U*^22^	*U*^33^	*U*^12^	*U*^13^	*U*^23^
In1	0.0318 (3)	0.0236 (3)	0.0380 (3)	0.00444 (17)	−0.01046 (18)	−0.00754 (18)
Co1	0.0216 (4)	0.0219 (4)	0.0252 (4)	0.0017 (3)	−0.0058 (3)	−0.0063 (3)
O1	0.042 (2)	0.025 (2)	0.031 (2)	0.0068 (18)	−0.0109 (18)	−0.0073 (17)
O2	0.068 (3)	0.030 (2)	0.031 (2)	0.011 (2)	−0.019 (2)	0.0007 (18)
O3	0.043 (2)	0.022 (2)	0.032 (2)	0.0033 (18)	−0.0167 (18)	−0.0064 (17)
O4	0.043 (2)	0.021 (2)	0.037 (2)	−0.0027 (18)	−0.0084 (18)	−0.0067 (17)
O5	0.027 (2)	0.030 (2)	0.066 (3)	0.0050 (18)	−0.013 (2)	−0.020 (2)
O6	0.030 (2)	0.035 (2)	0.075 (3)	0.0086 (19)	−0.007 (2)	−0.019 (2)
O7	0.028 (2)	0.025 (2)	0.052 (3)	0.0011 (17)	−0.0104 (18)	−0.0103 (19)
O8	0.045 (3)	0.038 (3)	0.082 (4)	0.006 (2)	−0.019 (2)	−0.037 (3)
O9	0.029 (2)	0.051 (3)	0.065 (3)	0.003 (2)	−0.001 (2)	−0.002 (2)
O10	0.044 (3)	0.050 (3)	0.064 (3)	0.008 (2)	−0.001 (2)	−0.008 (3)
O11	0.080 (4)	0.052 (3)	0.049 (3)	0.007 (3)	−0.019 (3)	0.015 (3)
O12	0.094 (4)	0.049 (3)	0.066 (4)	−0.001 (3)	−0.037 (3)	0.002 (3)
N1	0.029 (3)	0.037 (3)	0.044 (3)	0.000 (2)	−0.006 (2)	−0.019 (2)
N2	0.036 (3)	0.043 (3)	0.031 (3)	0.003 (2)	−0.009 (2)	−0.009 (2)
N3	0.032 (3)	0.033 (3)	0.041 (3)	0.001 (2)	−0.007 (2)	−0.012 (2)
N4	0.034 (3)	0.032 (3)	0.037 (3)	0.002 (2)	−0.008 (2)	−0.008 (2)
N5	0.028 (2)	0.026 (3)	0.037 (3)	0.003 (2)	−0.004 (2)	−0.007 (2)
N6	0.039 (3)	0.024 (3)	0.040 (3)	0.003 (2)	−0.013 (2)	−0.004 (2)
C1	0.111 (7)	0.073 (6)	0.038 (4)	−0.031 (5)	−0.019 (4)	−0.012 (4)
C2	0.097 (7)	0.065 (5)	0.050 (5)	0.013 (5)	−0.037 (4)	−0.024 (4)
C3	0.029 (3)	0.051 (4)	0.053 (4)	−0.009 (3)	−0.010 (3)	−0.017 (3)
C4	0.034 (3)	0.034 (3)	0.042 (4)	−0.007 (3)	−0.002 (3)	−0.006 (3)
C5	0.039 (3)	0.034 (3)	0.033 (3)	−0.001 (3)	−0.002 (3)	−0.002 (3)
C6	0.045 (4)	0.032 (3)	0.031 (3)	−0.006 (3)	−0.003 (3)	−0.001 (3)
C7	0.033 (3)	0.025 (3)	0.032 (3)	0.005 (2)	−0.007 (2)	−0.008 (2)
C8	0.026 (3)	0.023 (3)	0.022 (3)	−0.001 (2)	−0.006 (2)	−0.005 (2)
C9	0.030 (3)	0.030 (3)	0.048 (4)	0.002 (3)	−0.008 (3)	−0.013 (3)
C10	0.030 (3)	0.028 (3)	0.048 (4)	−0.001 (3)	−0.012 (3)	−0.005 (3)
C11	0.027 (3)	0.056 (5)	0.046 (4)	0.007 (3)	−0.003 (3)	0.001 (3)
C12	0.057 (5)	0.060 (5)	0.046 (4)	−0.008 (4)	−0.015 (4)	0.012 (4)
O2W	0.076 (4)	0.049 (3)	0.046 (3)	0.009 (3)	−0.028 (2)	−0.018 (2)
O1W	0.066 (3)	0.078 (4)	0.038 (3)	−0.022 (3)	0.005 (2)	−0.013 (3)

### Geometric parameters (Å, °)

**Table:**

In1—O11	2.140 (5)	N4—C4	1.485 (7)
In1—O9	2.143 (4)	N4—H4A	0.9000
In1—O7	2.160 (4)	N4—H4B	0.9000
In1—O5	2.164 (4)	N5—C5	1.489 (8)
In1—O3	2.171 (4)	N5—H5A	0.9000
In1—O1	2.203 (4)	N5—H5B	0.9000
Co1—N3	1.960 (5)	N6—C6	1.489 (8)
Co1—N6	1.970 (5)	N6—H6A	0.9000
Co1—N2	1.976 (5)	N6—H6B	0.9000
Co1—N4	1.981 (5)	C1—C2	1.439 (11)
Co1—N5	1.986 (5)	C1—H1C	0.9700
Co1—N1	1.997 (5)	C1—H1D	0.9700
O1—C7	1.292 (7)	C2—H2C	0.9700
O2—C7	1.230 (7)	C2—H2D	0.9700
O3—C8	1.280 (6)	C3—C4	1.507 (9)
O4—C8	1.239 (6)	C3—H3C	0.9700
O5—C9	1.259 (7)	C3—H3D	0.9700
O6—C9	1.238 (7)	C4—H4C	0.9700
O7—C10	1.290 (7)	C4—H4D	0.9700
O8—C10	1.232 (7)	C5—C6	1.524 (8)
O9—C11	1.286 (8)	C5—H5C	0.9700
O10—C11	1.229 (8)	C5—H5D	0.9700
O11—C12	1.247 (9)	C6—H6C	0.9700
O12—C12	1.262 (10)	C6—H6D	0.9700
N1—C1	1.509 (9)	C7—C8	1.565 (7)
N1—H1A	0.9000	C9—C10	1.587 (8)
N1—H1B	0.9000	C11—H11A	0.9300
N2—C2	1.459 (9)	C12—H12A	0.9300
N2—H2A	0.9000	O2W—H2WA	0.5459
N2—H2B	0.9000	O2W—H2WB	0.5460
N3—C3	1.493 (8)	O1W—H1WA	0.8043
N3—H3A	0.9000	O1W—H1WB	0.8031
N3—H3B	0.9000		
			
O11—In1—O9	88.9 (2)	Co1—N5—H5B	109.9
O11—In1—O7	110.48 (19)	H5A—N5—H5B	108.3
O9—In1—O7	86.65 (17)	C6—N6—Co1	109.6 (4)
O11—In1—O5	94.8 (2)	C6—N6—H6A	109.8
O9—In1—O5	163.60 (18)	Co1—N6—H6A	109.8
O7—In1—O5	77.07 (15)	C6—N6—H6B	109.8
O11—In1—O3	82.94 (18)	Co1—N6—H6B	109.8
O9—In1—O3	104.71 (18)	H6A—N6—H6B	108.2
O7—In1—O3	162.86 (16)	C2—C1—N1	111.7 (6)
O5—In1—O3	91.61 (16)	C2—C1—H1C	109.3
O11—In1—O1	157.73 (19)	N1—C1—H1C	109.3
O9—In1—O1	90.38 (17)	C2—C1—H1D	109.3
O7—In1—O1	91.69 (15)	N1—C1—H1D	109.3
O5—In1—O1	92.12 (16)	H1C—C1—H1D	107.9
O3—In1—O1	75.71 (14)	C1—C2—N2	109.6 (6)
N3—Co1—N6	89.5 (2)	C1—C2—H2C	109.8
N3—Co1—N2	91.9 (2)	N2—C2—H2C	109.8
N6—Co1—N2	175.7 (2)	C1—C2—H2D	109.8
N3—Co1—N4	85.3 (2)	N2—C2—H2D	109.8
N6—Co1—N4	94.4 (2)	H2C—C2—H2D	108.2
N2—Co1—N4	89.8 (2)	N3—C3—C4	108.0 (5)
N3—Co1—N5	173.0 (2)	N3—C3—H3C	110.1
N6—Co1—N5	85.1 (2)	C4—C3—H3C	110.1
N2—Co1—N5	93.7 (2)	N3—C3—H3D	110.1
N4—Co1—N5	90.6 (2)	C4—C3—H3D	110.1
N3—Co1—N1	91.9 (2)	H3C—C3—H3D	108.4
N6—Co1—N1	90.6 (2)	N4—C4—C3	106.1 (5)
N2—Co1—N1	85.3 (2)	N4—C4—H4C	110.5
N4—Co1—N1	174.2 (2)	C3—C4—H4C	110.5
N5—Co1—N1	92.7 (2)	N4—C4—H4D	110.5
C7—O1—In1	113.2 (3)	C3—C4—H4D	110.5
C8—O3—In1	114.3 (3)	H4C—C4—H4D	108.7
C9—O5—In1	114.6 (4)	N5—C5—C6	106.6 (5)
C10—O7—In1	113.9 (4)	N5—C5—H5C	110.4
C11—O9—In1	124.1 (5)	C6—C5—H5C	110.4
C12—O11—In1	122.2 (5)	N5—C5—H5D	110.4
C1—N1—Co1	107.6 (4)	C6—C5—H5D	110.4
C1—N1—H1A	110.2	H5C—C5—H5D	108.6
Co1—N1—H1A	110.2	N6—C6—C5	105.9 (5)
C1—N1—H1B	110.2	N6—C6—H6C	110.6
Co1—N1—H1B	110.2	C5—C6—H6C	110.6
H1A—N1—H1B	108.5	N6—C6—H6D	110.6
C2—N2—Co1	110.7 (4)	C5—C6—H6D	110.6
C2—N2—H2A	109.5	H6C—C6—H6D	108.7
Co1—N2—H2A	109.5	O2—C7—O1	126.1 (5)
C2—N2—H2B	109.5	O2—C7—C8	118.4 (5)
Co1—N2—H2B	109.5	O1—C7—C8	115.5 (5)
H2A—N2—H2B	108.1	O4—C8—O3	125.3 (5)
C3—N3—Co1	108.8 (4)	O4—C8—C7	118.6 (5)
C3—N3—H3A	109.9	O3—C8—C7	116.1 (4)
Co1—N3—H3A	109.9	O6—C9—O5	125.9 (6)
C3—N3—H3B	109.9	O6—C9—C10	118.0 (5)
Co1—N3—H3B	109.9	O5—C9—C10	116.1 (5)
H3A—N3—H3B	108.3	O8—C10—O7	125.8 (5)
C4—N4—Co1	110.4 (4)	O8—C10—C9	118.9 (5)
C4—N4—H4A	109.6	O7—C10—C9	115.3 (5)
Co1—N4—H4A	109.6	O10—C11—O9	126.7 (6)
C4—N4—H4B	109.6	O10—C11—H11A	116.7
Co1—N4—H4B	109.6	O9—C11—H11A	116.7
H4A—N4—H4B	108.1	O11—C12—O12	124.2 (7)
C5—N5—Co1	109.0 (4)	O11—C12—H12A	117.9
C5—N5—H5A	109.9	O12—C12—H12A	117.9
Co1—N5—H5A	109.9	H2WA—O2W—H2WB	109.4
C5—N5—H5B	109.9	H1WA—O1W—H1WB	98.5

### Hydrogen-bond geometry (Å, °)

**Table:**

*D*—H···*A*	*D*—H	H···*A*	*D*···*A*	*D*—H···*A*
N1—H1A···O7^i^	0.90	2.22	3.019 (7)	148
N1—H1B···O6^ii^	0.90	2.25	2.981 (7)	139
N2—H2A···O1W^iii^	0.90	2.02	2.857 (7)	155
N2—H2B···O4^iv^	0.90	2.14	3.017 (6)	166
N3—H3A···O2W^v^	0.90	2.23	3.017 (7)	146
N3—H3B···O6^ii^	0.90	2.17	3.002 (7)	154
N3—H3B···O8^ii^	0.90	2.50	3.153 (7)	130
N4—H4A···O2	0.90	2.11	2.915 (6)	148
N4—H4B···O10^iv^	0.90	2.33	3.102 (7)	144
N4—H4B···O2^iv^	0.90	2.53	3.166 (7)	128
N5—H5A···O8^i^	0.90	2.16	2.986 (6)	153
N5—H5B···O4^iv^	0.90	2.27	3.022 (6)	141
N5—H5B···O2^iv^	0.90	2.28	3.060 (6)	145
N6—H6A···O1	0.90	2.05	2.917 (7)	161
N6—H6B···O6^ii^	0.90	2.19	3.024 (7)	154
O2W—H2WA···O3^vi^	0.55	2.36	2.889 (6)	163
O2W—H2WB···O12^vii^	0.55	2.22	2.751 (7)	166
O1W—H1WA···O2W	0.80	2.03	2.821 (7)	169
O1W—H1WB···O4	0.80	2.09	2.836 (6)	156

Symmetry codes: (i) −*x*+2, −*y*+2, −*z*+1; (ii) −*x*+1, −*y*+2, −*z*+1; (iii) *x*, *y*, *z*+1; (iv) −*x*+2, −*y*+1, −*z*+1; (v) −*x*+1, −*y*+1, −*z*+1; (vi) −*x*+1, −*y*+1, −*z*; (vii) *x*, *y*−1, *z*.
